# Ability of NAD and Sirt1 to epigenetically suppress albuminuria

**DOI:** 10.1007/s10157-024-02502-w

**Published:** 2024-04-08

**Authors:** Kazuhiro Hasegawa, Masanori Tamaki, Eriko Shibata, Taizo Inagaki, Masanori Minato, Sumiyo Yamaguchi, Ikuko Shimizu, Shinji Miyakami, Miho Tada, Shu Wakino

**Affiliations:** Department of Nephrology, Tokushima University Graduate School of Biomedical Sciences, 3-18-15 Kuramoto-cho, Tokushima, 770-8503 Japan

**Keywords:** Sirtuin 1, Nicotinamide mononucleotide, Claudin-1

## Abstract

The time for diabetic nephropathy (DN) to progress from mild to severe is long. Thus, methods to continuously repress DN are required to exert long-lasting effects mediated through epigenetic regulation. In this study, we demonstrated the ability of nicotinamide adenine dinucleotide (NAD) and its metabolites to reduce albuminuria through Sirt1- or Nampt-dependent epigenetic regulation. We previously reported that proximal tubular Sirt1 was lowered before glomerular Sirt1. Repressed glomerular Sirt1 was found to epigenetically elevate Claudin-1. In addition, we reported that proximal tubular Nampt deficiency epigenetically augmented TIMP-1 levels in Sirt6-mediated pathways, leading to type-IV collagen deposition and diabetic fibrosis. Altogether, we propose that the Sirt1/Claudin-1 axis may be crucial in the onset of albuminuria at the early stages of DN and that the Nampt/Sirt6/TIMP-1 axis promotes diabetic fibrosis in the middle to late stages of DN. Finally, administration of NMN, an NAD precursor, epigenetically potentiates the regression of the onset of DN to maintain Sirt1 and repress Claudin-1 in podocytes, suggesting the potential use of NAD metabolites as epigenetic medications for DN.

## Introduction

Sirtuins (Sirts) are deacetylating enzymes that epigenetically affect the expression of many proteins by deacetylating histones and transcriptional factors. Among seven Sirt isoforms, we have previously revealed the role of Sirtuin1 (Sirt1), an Sirt isoform, in diabetic nephropathy (DN), which is a diabetes-induced kidney damage, by assessing Sirt1-gene-engineered mice [1]. First, proximal tubular Sirt1 levels decreased, followed by decreased Sirt1 levels in podocytes [1]. We named these changes as tubuloglomerular interplay [2]. In glomeruli, claudin-1, an important protein of the tight junction, is localized in parietal epithelial cells (PECs) under normal conditions. However, in diabetic conditions, claudin-1 is abnormally detected in podocytes, which is considered an ectopic expression [2]. This condition occurs continuously as DN progresses and is epigenetically regulated by activated Dnmt1 because of decreased Sirt1 levels [1]. Sirt1 activity depends on nicotinamide adenine dinucleotide (NAD) levels. In addition, we recently demonstrated that nicotinamide mononucleotide (NMN), a precursor of NAD, efficiently boosts renal NAD levels rather than NAD itself in an epigenetic manner [3]. Therefore, in the initial step, where Sirt1 expression in the proximal tubules was downregulated, NMN and NAD levels were lowered in the proximal tubules. Next, Sirt1 activity in podocytes was repressed, and NMN and NAD levels were diminished in podocytes. NMN administration activated Sirt1 levels, which suppressed albuminuria by epigenetically suppressing claudin-1 ectopic expression. Overall, our findings suggest that the NMN–Sirt1 axis is a potential target for epigenetic therapeutic approaches in DN to prevent the decline of NMN, NAD, and Sirt1 levels in the kidneys.

## Antiaging methods

**Fig. 1:**
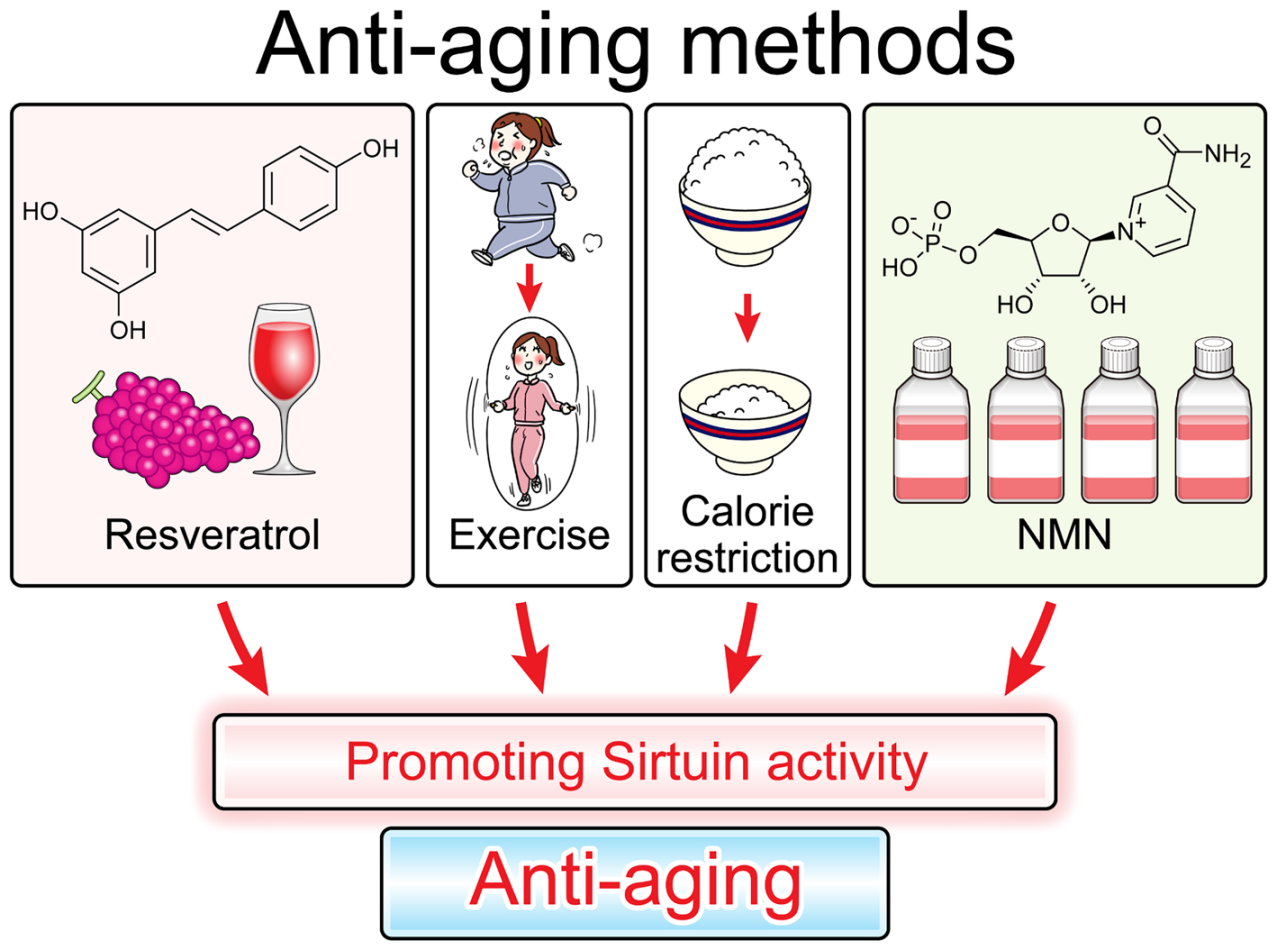
Resveratrol, exercise, calorie restriction, and NMN administration promote Sirtuin activity, which may subsequently promote antiaging

Consistent with recent reports [4], our findings show that several factors may increase lifespan. Although numerous methods have been suggested, resveratrol administration, exercise, calorie restriction, and NMN administration reportedly activate Sirt, yielding longevity [5]. As for the oral administration of NAD, it is easily degraded by the intestinal microbiota [6]. It is unclear whether intravenous or intraperitoneal administration of NAD is effective; however, NAD is more easily degraded under atmospheric conditions such as high-temperature environments. Thus, NMN is regarded as a favorite tool for elevating NAD levels. Figure 1 outlines the antiaging methods via Sirt.

## Upstream and downstream regions of Sirt

**Fig. 2:**
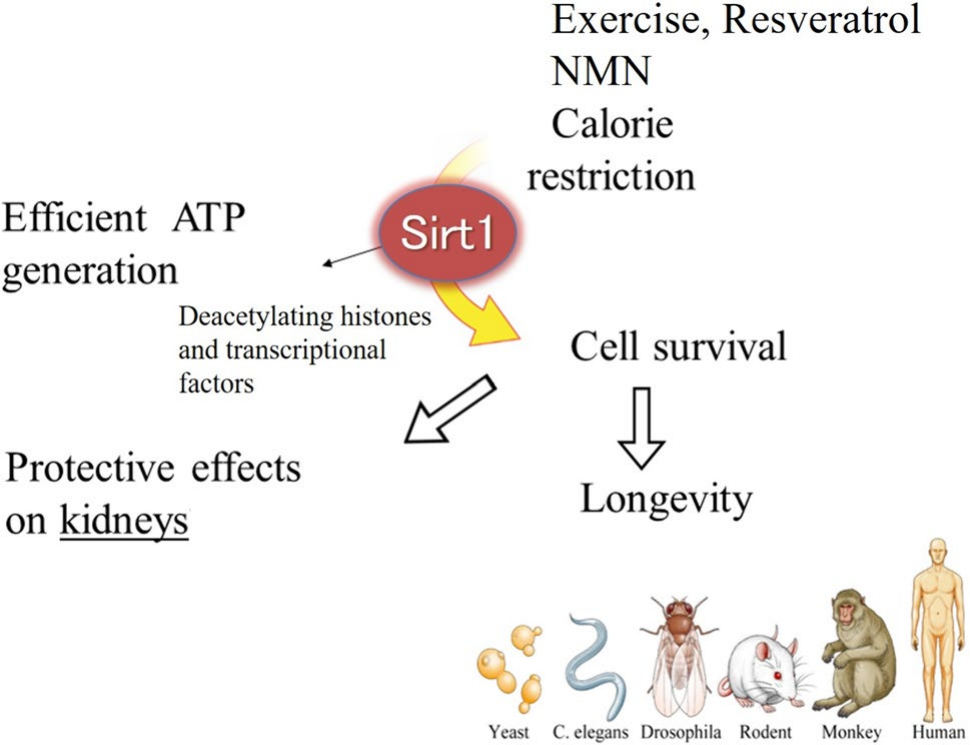
Upstream and downstream regions of Sirt1. Calorie restriction elevates Sirt1 activity, inducing efficient ATP generation in each cell. Sirt-mediated cell survival in the whole body increases longevity. These effects also accompany organ protection throughout the body. We have investigated whether Sirt1 protects against kidney injury

In the upstream region of Sirt, there are calorie restriction and other factors (Fig. 1). Sirt efficiently generates adenosine triphosphate (ATP) by deacetylating important histones and transcriptional factors downstream of Sirt. It is responsible for cell survival and the longevity of organisms (Fig. 2). Furthermore, Sirt has a protective effect on each organelle. Therefore, we aimed to determine whether Sirt could exert a protective effect on kidneys, focusing on Sirt1 [6].

## Sirt-mediated organelle protection

**Fig. 3:**
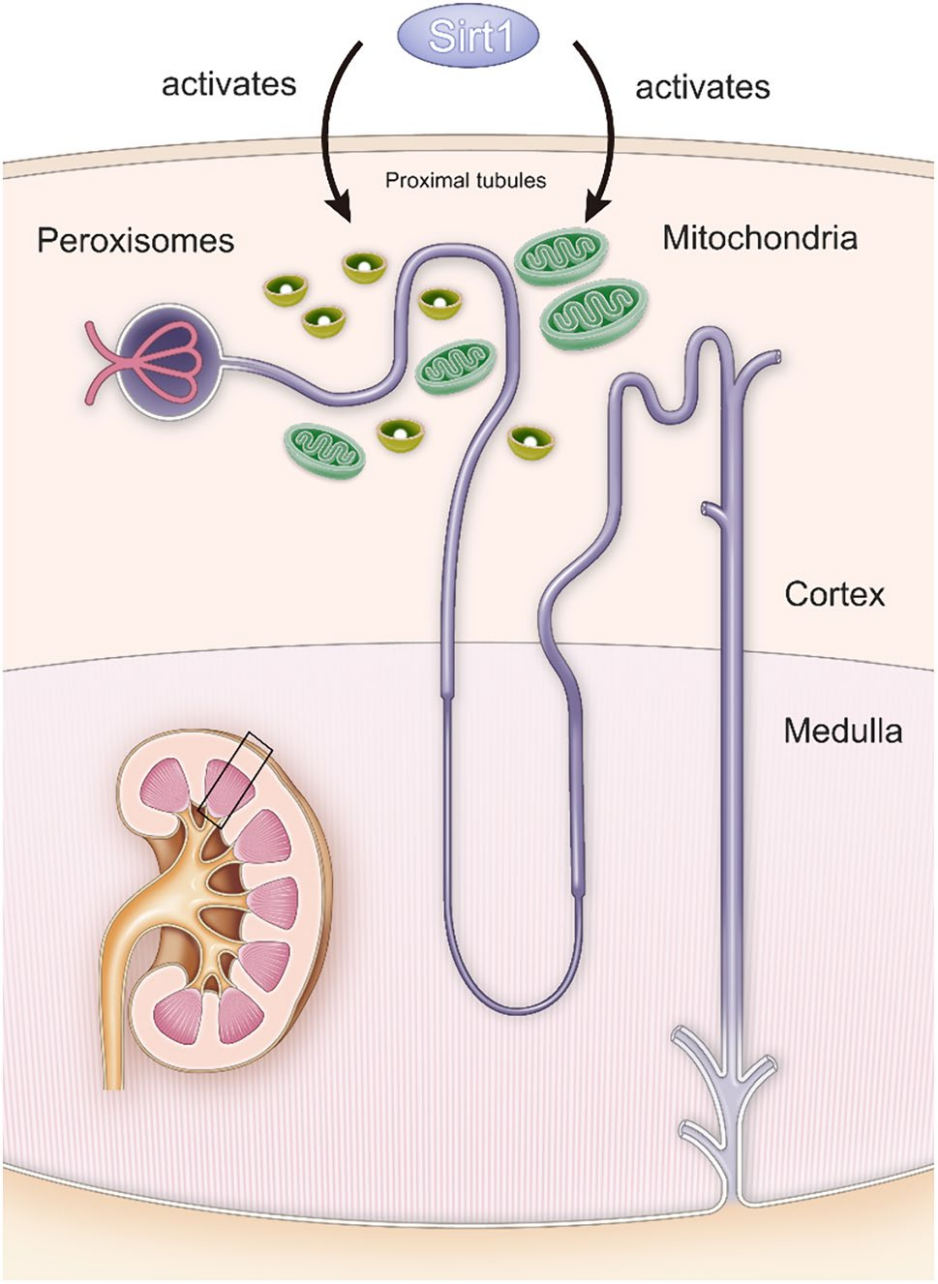
Sirt1 mediates organelle protection. Proximal tubules require high amounts of ATP to reabsorb the necessary substances in the urine. Therefore, proximal tubules contain abundant mitochondria and peroxisomes. Sirt1 reportedly activates mitochondrial and peroxisomal functions. Thus, Sirt1 is presumed to play an important role in the proximal tubules

Sirt contributes to ATP production, suggesting that Sirt also activates mitochondria and peroxisomes, which are ATP generators that orchestrate glycolysis and fatty acid oxidation (FAO) in cells. The kidneys contain large amounts of peroxisomes and mitochondria, which are distributed in the proximal tubules to produce ATP and preserve the energy required for reabsorbing many nutrients in urine, such as glucose and amino acids. Thus, it is presumed that renal Sirt plays a pivotal role in proximal tubular cells (Fig. 3). Among the seven Sirt isoforms, we focused on Sirt1 in the kidneys because it affects mitochondrial and peroxisomal function.

## NAD-related metabolic map

**Fig. 4:**
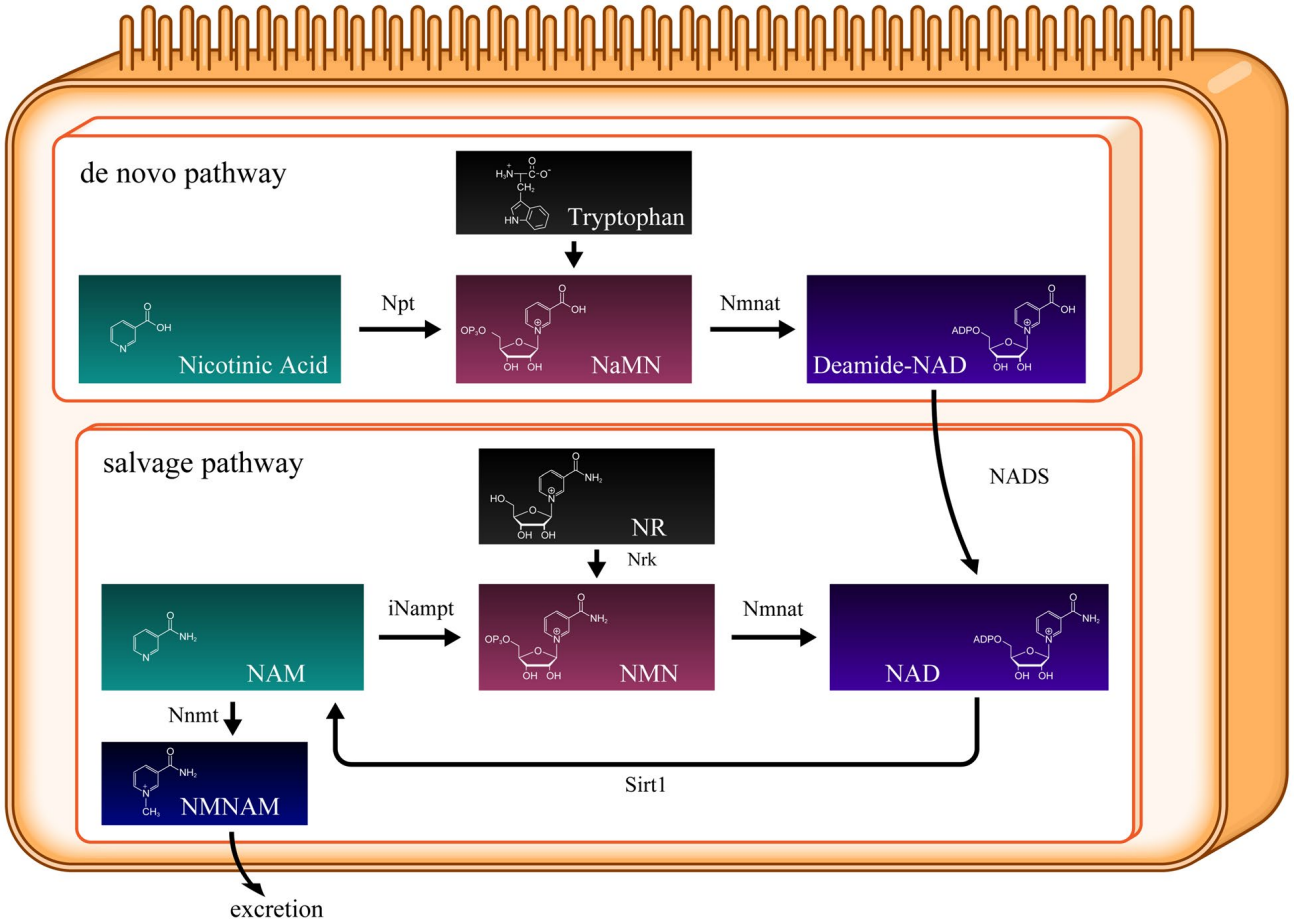
NAD-related metabolic map. NAD synthesis occurs via two pathways: de novo and salvage pathways. NAM, nicotinamide; Npt, nicotinic acid phosphoribosyltransferase; 5′-NT, 5′-nucleotidase; NaMN, nicotinic acid mononucleotide; NR, nicotinamide riboside; NMN, nicotinamide mononucleotide; iNampt, intracellular NAM phosphoribosyl transferase; NrK, nicotinamide riboside kinases; Nmnat, nicotinamide mononucleotide adenylyl transferases; NADS, NAD synthetase. Nicotinamide N-methyltransferase (Nnmt) converts NAM to N1-methylniacinamide (NMNAM), which is excreted in urine

In the human body, there are two NAD-generating pathways: the de novo and salvage pathways. In the de novo pathway, tryptophan, which is mainly derived from food intake, synthesizes new NAD. In the salvage pathway, NAD is recycled via three chemical reactions. In this pathway, Sirt1 deacetylates many transcription factors and histones that require NAD. In the Sirt1-mediated deacetylation reaction, NAD is consumed and converted to nicotinamide (NAM) (Fig. 4**)**. As Sirt1 is activated, it aggressively deacetylates histones and transcriptional factors, significantly progressing NAD consumption. The two enzymes—nicotinamide phosphoribosyltransferase (Nampt) and NMN adenylyltransferase (Nmnat)—facilitate NAD regeneration. Nampt has two isoforms: intracellular Nampt (iNampt) and extracellular Nampt (eNampt). iNampt is responsible for generating NMN inside each cell. eNampt is secreted from some cells and transferred mainly via the bloodstream to other cells. However, the functions of eNampt and the difference between iNampt and eNampt remain unknown [7]. Nmnat comprises three isoforms: Nmnat1, 2, and 3. Nmnat1 is nuclear, Nmnat2 is cytoplasmic, and Nmnat3 is localized in the Golgi apparatus [8]. iNampt catalyzes NAM into NMN, whereas Nmnat produces NAD from NMN.

## Changes in Sirt1 and NMN in the very early stages of DN

**Fig. 5:**
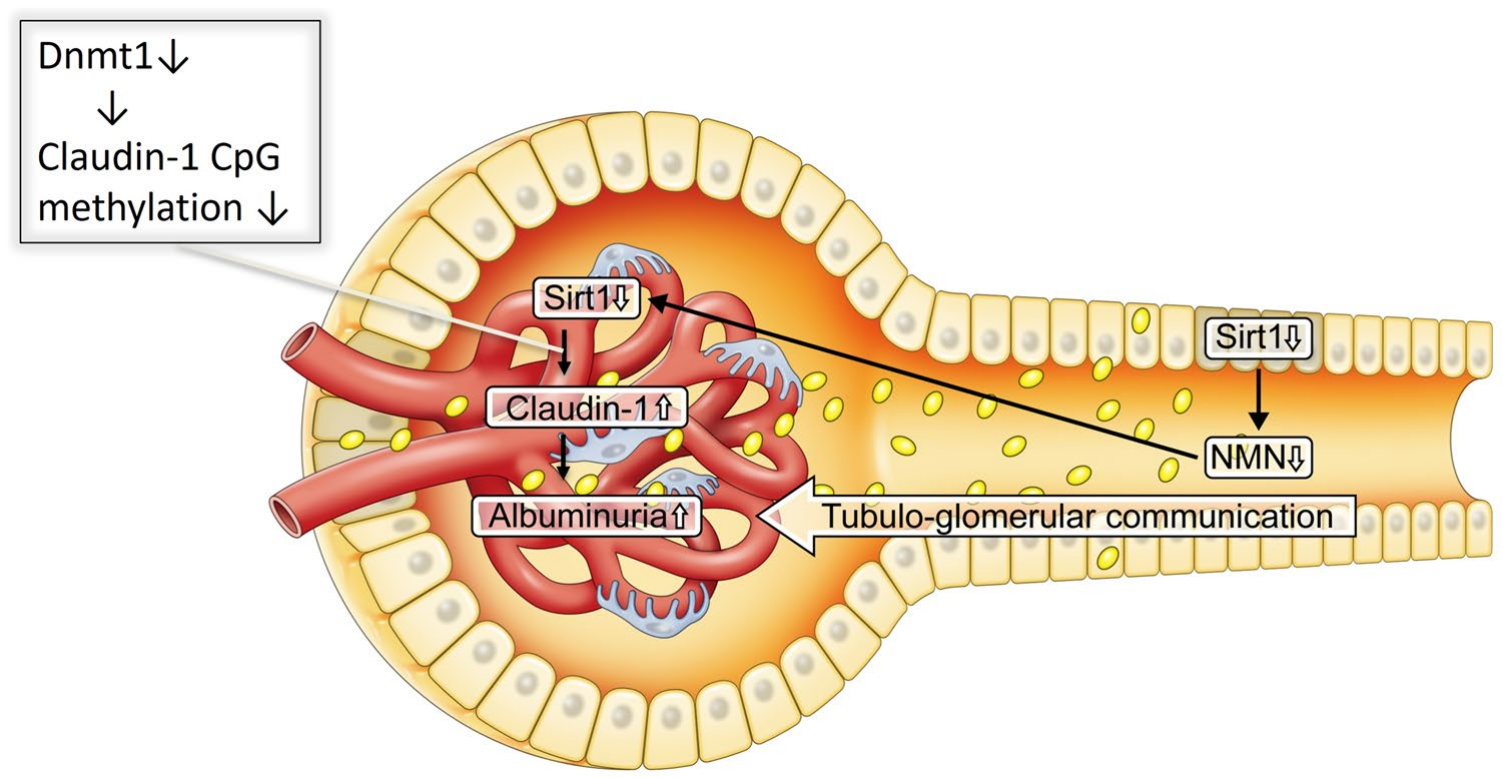
Schema depicting proximal tubule–podocyte communication. High glucose exposure primarily downregulates proximal tubular Sirt1 levels. This reduces NMN and podocyte Sirt1 levels, accompanied by Claudin-1 induction and albuminuria

Sirt1 expression in the proximal tubule is primarily downregulated before podocytic changes in Sirt1 levels, consistent with the decline in NMN levels in the whole kidney. Subsequently, the decreased NMN levels further mitigate the Sirt1 levels in podocytes. In the NAD salvage pathway, NMN is located upstream of NAD and Sirt1. Because the salvage pathway is a circuit pathway, NMN is also localized upstream and downstream of Sirt1. We previously demonstrated that NMN is distributed from the proximal tubules to the podocytes using fluorescence-tagged NMN [1]. We named this communication proximal tubule–podocyte communication (Fig. 5). However, this communication is disrupted in DN because of NMN deficiency.

Reduced Sirt1 levels in podocytes induce the ectopic expression of claudin-1 in podocytes via epigenetic regulatory mechanisms, as described in the following section. Claudin-1 is detectable in PECs and constructs tight junctions in the Bowman’s capsule, preventing urine leakage under normal conditions [8]. Conversely, Claudin-1 is newly expressed in podocytes induced by reduced Sirt1 expression under DN. Gong et al. created transgenic (TG) mice overexpressing claudin-1 in podocytes, representing albuminuria because of podocyte injury in association with downregulated nephrin and podocin [9]. Because of our findings regarding the role of the Sirt1/Claudin-1 axis on podocyte function, many other reports have shown that depressed Sirt1 and amplified Claudin-1 were detectable in damaged podocytes in DN [10, 11] and in other kidney diseases such as focal segmental glomerulosclerosis [12, 13] and hypertension-induced glomerular sclerosis [14]. In this regard, the regulatory mechanism by which repressed Sirt1 augmented Claudin-1 is considered significant for establishing the potential of diagnostic markers and/or therapeutic targets of the Sirt1/Claudin-1 axis in kidney diseases [15, 16]. This is explained in more detail in the following section.

## Epigenetic regulation of claudin-1 expression in podocytes by Sirt1

**Fig. 6:**
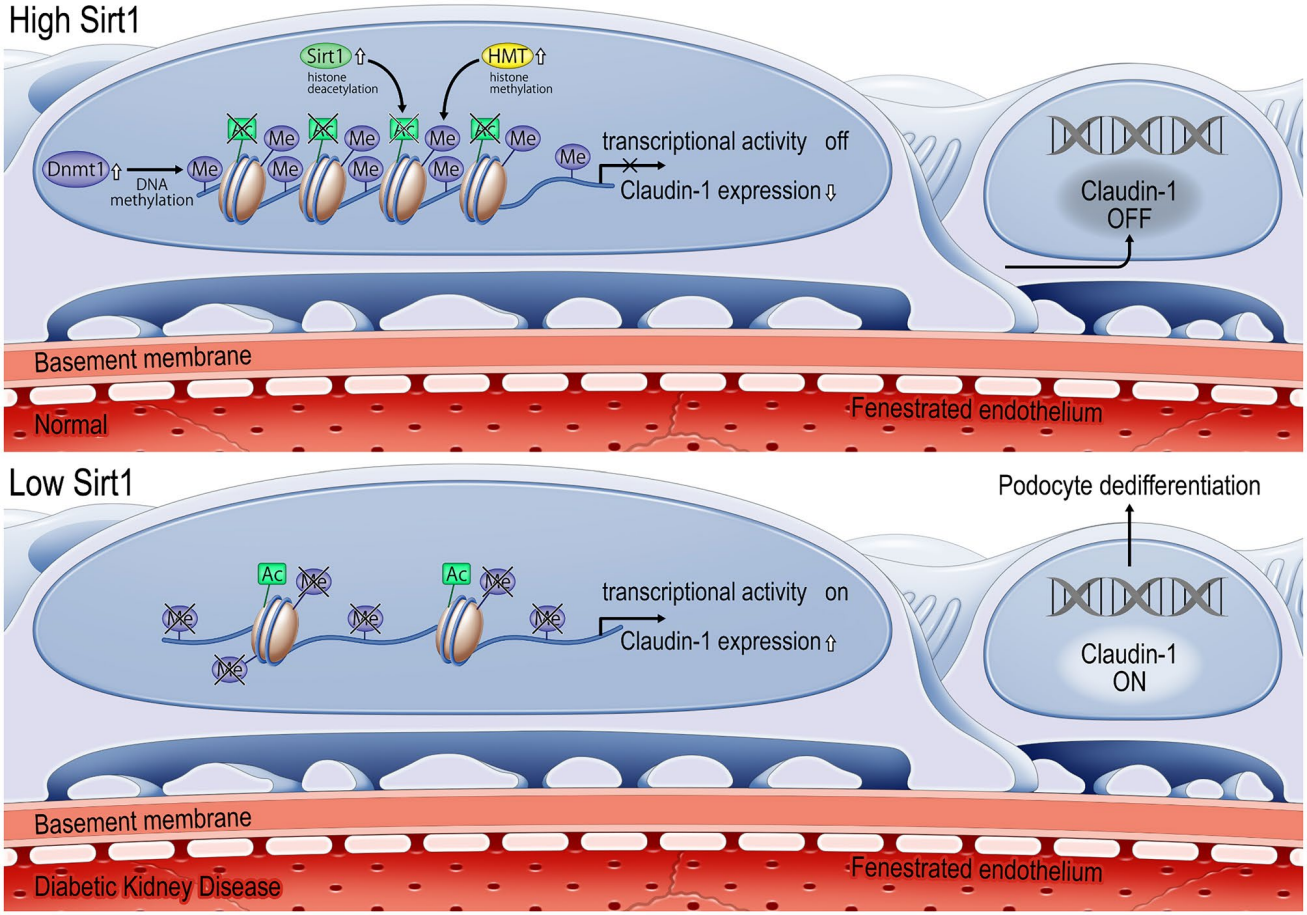
Epigenetic regulation of claudin-1 expression in podocytes by Sirt1. Under normal glucose conditions, retained Sirt1 deacetylates H3 and H4 histones, resulting in H3K9 histone methylation and Dnmt1 activation. H3K9, histone H3 Lys^9^; HMT, histone methyltransferases; ME, methyl-; DNMT, DNA methyltransferases; CG, CpG islands

We examined the molecular mechanisms by which Sirt1 alters claudin-1 expression in podocytes. In our experimental results, we identified new epigenetic mechanisms in podocytes (Fig. 6). Under basal conditions, Sirt1 levels are highly retained. Sirt1 deacetylates histones H3 and H4, leading to histone H3K9 methylation. This also causes DNA methylation of the CpG islands in the claudin-1 gene induced by DNA methyltransferase 1 (Dnmt1), which contributes to the repression of claudin-1 expression. Furthermore, Sirt1 reportedly activates Dnmt1 [17]. Under diabetic conditions, Sirt1 levels were subdued, increasing H3 and H4 acetylation and decreasing H3K9 and Dnmt1-induced CpG methylation of the claudin-1 promoter region to induce its mRNA expression and concomitant protein levels.

## Effect of Nampt knockdown on extracellular matrix deposition

**Fig. 7:**
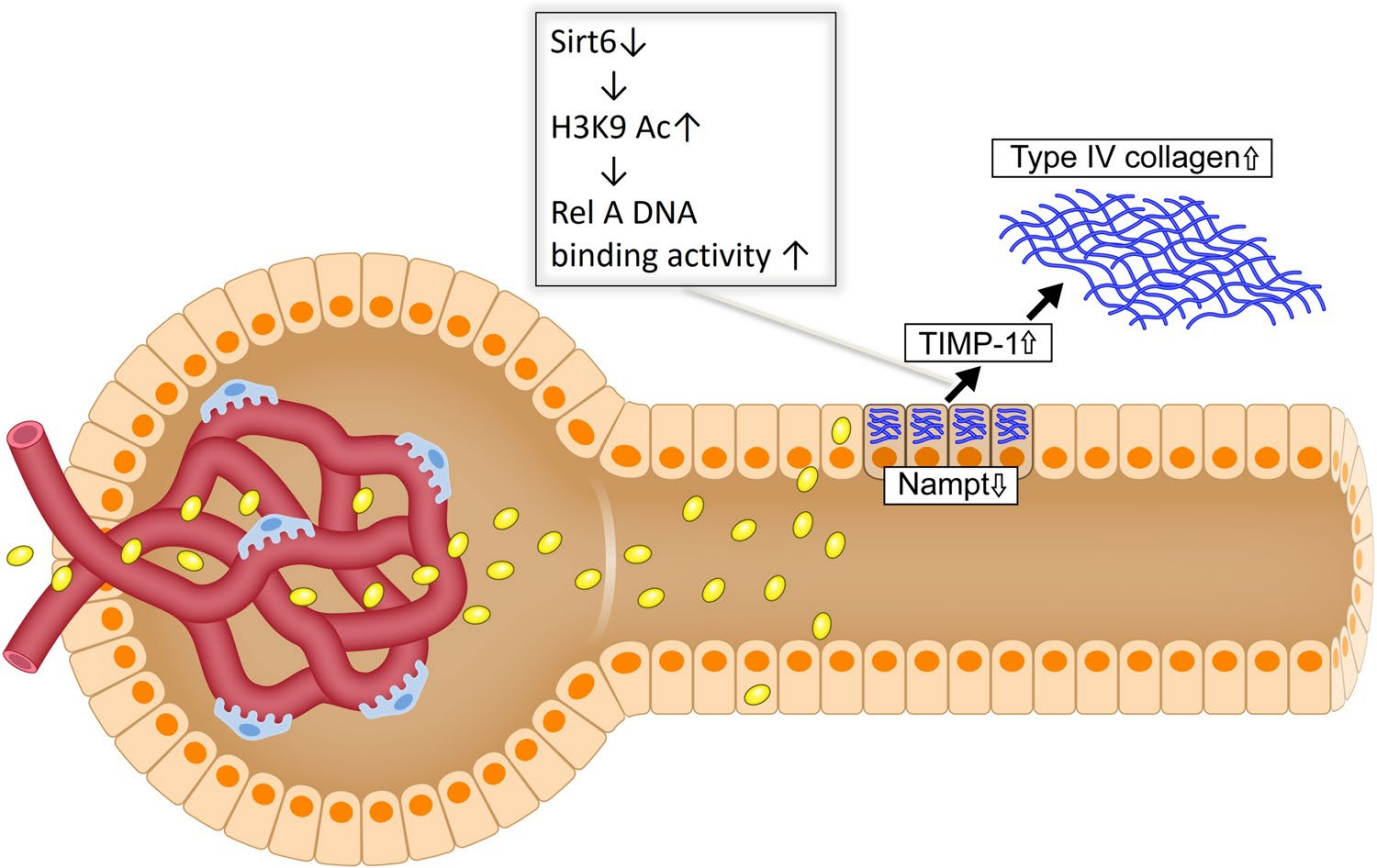
Scheme depicting the mechanism of Nampt-related type-IV collagen deposition in DN. In DN, a decrease in Nampt suppresses Sirt6 levels. Augmented acetylation of H3K9 mediated by downregulated Sirt6 escalates TIMP-1 expression because RelA attaches to the RelA-binding site in the promoter of TIMP-1. Elevation of TIMP-1 induces type-IV collagen production

Nicotinamide phosphoribosyl transferase (Nampt) is considered indispensable for renal pathophysiology because it produces NMN. To investigate the specific role of Nampt in kidneys (Fig. 7), we created and assessed the phenotypes of PT-specific Nampt-overexpressing TG mice and PT-specific Nampt-conditional knockout (Nampt CKO) mice [18]. These phenotypic changes showed that the Nampt CKO mice mitigated one of the sirtuins, particularly Sirt6, rather than Sirt1, with elevated levels of H3K9Ac and increased Timp-1 expression. Timp-1 overexpression prompted type-IV collagen deposition, similar to the changes in DN. Conversely, the TG mice were protected against streptozotocin or db/db-induced diabetic extracellular matrix depositions such as type-IV collagen accumulation. Thus, the Nampt-Sirt6 axis-related epigenetic changes in PTs are vital in fibrogenic extracellular matrix production in DN. However, the mechanisms by which Nampt knockout affects Sirt6 rather than Sirt1 require further investigation.

## Molecular mechanisms of dysfunctional proximal tubule–podocyte communication in DN

**Fig. 8:**
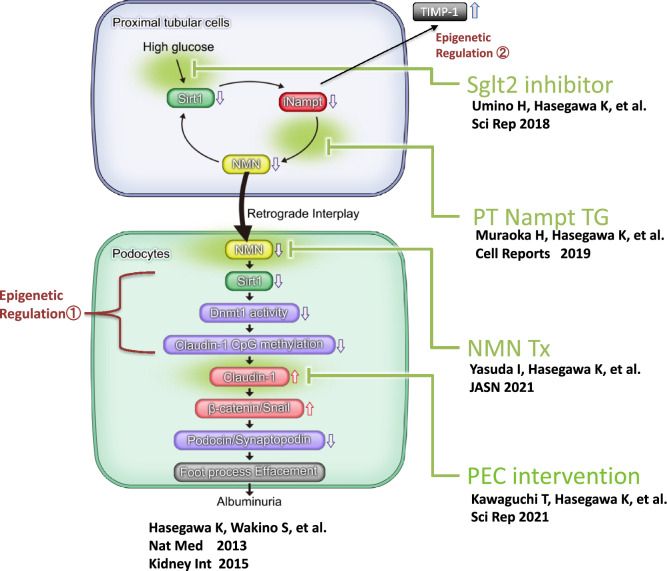
Molecular mechanisms of dysfunctional proximal tubule–podocyte communication in DN. Primal changes occurred in the proximal tubules, where proximal tubular Sirt1 is mitigated by the continuous high stimulation of glucose production mediated by Sglt2. Sglt2 inhibitors block this vicious intracellular signal flow. Sirt1 reduction leads to a decrease in Nampt and NMN levels, downregulating Sirt1 expression in podocytes. This is prohibited by Sirt1 and/or Nampt overexpression. Furthermore, Nampt knockdown also elevated TIMP-1 levels, triggering massive renal fibrosis, which is consistent with the initial stages of DN-induced renal fibrosis. NMN administration prevents NMN deficiency. Claudin-1 overexpression induces podocyte damage, which may be blocked by claudin-1 knockdown in PECs. Our previous findings suggest that epigenetic regulatory pathways are involved in Nampt deficiency-induced TIMP-1 elevation in PTs and Sirt1 defect-mediated Claudin-1 epitopic expression in podocytes

We recently discovered that sodium-glucose cotransporter-2 (SGLT2) is significantly involved in the decreased Sirt1 levels in proximal tubules. Conversely, SGLT2 inhibitors preserve Sirt1 expression [19]. We also found that the damaged proximal tubule–podocyte interaction is caused by the reduced levels of proximal tubular Sirt1 concomitant with decreased Nampt and NMN levels. NMN defects lead to the epitopic expression of Claudin-1 in podocytes. Our findings suggest that β-catenin/snail downstream of the epitopic expression of Claudin-1 is relevant in podocyte damage, as shown in Fig. 8**.**

## Conceptions and perspectives

In DN, SGLT2 contributes to the reduction of Sirt1 expression. However, the detailed mechanisms underlying the decreased Sirt1 levels require further investigation. Previously, glucose was not metabolized in proximal tubules; however, recent research has shown the importance of aberrant renal glycolysis even in the renal cortex [20]. This suggests that proximal tubules use glucose as fuel under specific conditions, although FAO is the main source of ATP demand under normal conditions. Furthermore, we recently showed that renal gluconeogenesis may also protect against DN-induced mitochondrial damage in the proximal tubules [21]. In this study, we demonstrated that the gluconeogenetic enzyme, Pck1, is a key regulator of mitochondrial ribosomes. In addition, other reports support the importance of Pck1 in renal gluconeogenesis [22, 23]. Thus, proximal tubules have three metabolic systems, glycolysis, gluconeogenesis, and FAO, in addition to glucose reabsorption via GLUT2 and SGLT2. Excessively high levels of glucose possibly decrease the NAD/NADH ratio or NAD levels in proximal tubules, probably decreasing Sirt1, Nampt, and NMN levels [24]. Future studies are also required to determine whether such a hypothesis is correct in the kidneys. If these molecular mechanisms are determined, the roles of the two epigenetic regulatory mechanisms, whereby decreased Sirt1 regulates Claudin-1 in podocytes and mitigated Nampt increases TIMP-1 levels on new therapeutic targets in DN, may become more significant in the future. In addition, the use of Claudin-1-specific monoclonal antibodies in renal, hepatic, and lung fibrosis models showed clear antifibrotic effects, further indicating the role of Claudin-1 as a therapeutic target for tissue fibrosis across organs [25].

A variety of studies using human renal samples underlying the correlation between Nampt/Sirt6 /TIMP-1 and diabetic nephropathy have been reported, which includes the important roles of these molecules mainly in the middle to late stages of DN [26, 27]. However, it is undeniable that Nampt/Sirt6/TIMP-1 may play a role not only in the middle to late stages but also in the early stages of DN because Nampt, Sirt1, and Sirt6 are enzymes working in the same NAD salvage pathways. Further studies are needed to uncover whether Sirt1/Claudin-1 is important in the early stages and whether Nampt/Sirt6/TIMP-1 is significant in the middle to late stages of human diabetic nephropathy, similar to that in murine models.

## Expectation for the future

The authors have discussed the importance of NAD and Sirtuins within the context of tubule-glomerular crosstalk in diabetic nephropathy. However, there are cases of diabetic kidney disease (DKD) where patients do not present with overt albuminuria yet experience a decline in renal function. According to previous studies, NAD and sirtuins do not play a critical role in renal dysfunction without overt albuminuria, wherein hypertension and/or atherosclerosis-mediated glomerulosclerosis results in the elevation of serum creatinine independent of urinary albuminuria. There are three reasons that support this notion. First, previous reports have demonstrated that Sirtuin1-7-knockout mice did not develop hypertension or glomerulosclerosis phenotypes leading to DKD-mediated renal dysfunction without overt albuminuria [28]. In contrast, we speculated that proximal tubule-specific Sirt1-knockout mice demonstrated overt albuminuria [1]. Second, we also identified that proximal tubular Sirt1 was downregulated by SGLT2/GLUT2-mediated intracellular signals in DN, which is in accordance with the finding that NAD and sirtuins are more influenced by high glucose-induced stimulation than hypertension-induced glomerulosclerotic injuries [19]. Third, in patients with DN, Sirt1’s single nucleotide polymorphisms are significantly correlated with overt albuminuria [29]. However, further studies using human DKD samples without albuminuria are required to confirm that such conditions do not change NAD and Sirtuins unlike DN with albuminuria.

Although we have stated the significance of Nampt in proximal tubules, future studies are also needed to assess the significance of Nampt in podocytes. According to the immunostaining results of Nampt in murine kidneys, Nampt expression was not detected in podocytes under normal conditions and in diabetic kidneys [18]. Additionally, the human atlas (Tissue expression of NAMPT—Staining in the kidney—The Human Protein Atlas) also showed that Nampt proteins were poorly detected in glomeruli but exist in tubular regions. Consistent with these findings, podocyte-specific Nampt-knockout mice have not been reported. Moreover, other researchers have clarified the role of proximal tubular Nampt in other kidney injuries [30, 31] aside from DN. However, further studies using human kidney samples are also required to confirm that Nampt plays a key role in the proximal tubules despite poor expression of Nampt in podocytes.

We have also described that short-term, intensive administration of NMN in the early stages of diabetic nephropathy has long-lasting renal protective effects. On the other hand, the therapeutic efficacy of NMN in the middle to late stages of DN has not been reported. However, we demonstrated that proximal tubule-specific Nampt TG succeeded in mitigating DN-related renal fibrosis [18]. Additionally, NMN administration repressed AKI-induced renal fibrosis [32]. Overall, these findings suggest the potential of NMN/NAD supplementation for inhibiting renal fibrosis, which usually develops in the middle to late stages of DN. Furthermore, the effects of long-term NMN administration have been depicted in other disease models [33, 34]. Therefore, long-term NMN/NAD treatments may inhibit renal damage even in the middle to late stages of DN.

## References

[1] Hasegawa K, Wakino S, Simic P, Sakamaki Y, Minakuchi H, Fujimura K, Hosoya K, Komatsu M, Kaneko Y, Kanda T, Kubota E, Tokuyama H, Hayashi K, Guarente L, Itoh H (2013). Renal tubular Sirt1 attenuates diabetic albuminuria by epigenetically suppressing Claudin-1 overexpression in podocytes. Nat Med.

[2] Hasegawa K, Wakino S, Sakamaki Y, Muraoka H, Umino H, Minakuchi H, Yoshifuji A, Naitoh M, Shinozuka K, Futatsugi K, Urai H, Kanda T, Tokuyama H, Hayashi K, Itoh H (2016). Communication from tubular epithelial cells to podocytes through Sirt1 and nicotinic acid metabolism. Curr Hypertens Rev.

[3] Yasuda I, Hasegawa K, Sakamaki Y, Muraoka H, Kawaguchi T, Kusahana E, Ono T, Kanda T, Tokuyama H, Wakino S, Itoh H (2021). Pre-emptive short-term nicotinamide mononucleotide treatment in a mouse model of diabetic nephropathy. J Am Soc Nephrol.

[4] Morigi M, Perico L, Benigni A (2018). Sirtuins in renal health and disease. J Am Soc Nephrol.

[5] Hasegawa K, Wakino S, Yoshioka K, Tatematsu S, Hara Y, Minakuchi H, Sueyasu K, Washida N, Tokuyama H, Tzukerman M, Skorecki K, Hayashi K, Itoh H (2010). Kidney-specific overexpression of Sirt1 protects against acute kidney injury by retaining peroxisome function. J Biol Chem.

[6] Hasegawa K, Wakino S, Yoshioka K, Tatematsu S, Hara Y, Minakuchi H, Washida N, Tokuyama H, Hayashi K, Itoh H (2008). Sirt1 protects against oxidative stress-induced renal tubular cell apoptosis by the bidirectional regulation of catalase expression. Biochem Biophys Res Commun.

[7] Park JW, Roh E, Kang GM, Gil SY, Kim HK, Lee CH, Jang WH, Park SE, Moon SY, Kim SJ, Jeong SY, Park CB, Lim HS, Oh YR, Jung HN, Kwon O, Youn BS, Son GH, Min SH, Kim MS (2023). Circulating blood eNAMPT drives the circadian rhythms in locomotor activity and energy expenditure. Nat Commun.

[8] Gong Y, Hou J (2017). Claudins in barrier and transport function-the kidney. Pflugers Arch.

[9] Gong Y, Sunq A, Roth RA, Hou J (2017). Inducible expression of Claudin-1 in glomerular podocytes generates aberrant tight junctions and proteinuria through slit diaphragm destabilization. J Am Soc Nephrol.

[10] Samadi M, Aziz SG, Naderi R (2021). The effect of tropisetron on oxidative stress, SIRT1, FOXO3a, and claudin-1 in the renal tissue of STZ-induced diabetic rats. Cell Stress Chaperones.

[11] Bible E (2013). Diabetic nephropathy: Sirt1 attenuates diabetic albuminuria. Nat Rev Nephrol.

[12] Hasegawa K, Sakamaki Y, Tamaki M, Wakino S (2022). Nicotinamide mononucleotide ameliorates adriamycin-induced renal damage by epigenetically suppressing the NMN/NAD consumers mediated by Twist2. Sci Rep.

[13] Lopes-Gonçalves G, Costa-Pessoa JM, Pimenta R, Tostes AF, da Silva EM, Ledesma FL, Malheiros DMAC, Zatz R, Thieme K, Câmara NOS, Oliveira-Souza M (2023). Evaluation of glomerular sirtuin-1 and claudin-1 in the pathophysiology of nondiabetic focal segmental glomerulosclerosis. Sci Rep.

[14] Martinez-Arroyo O, Ortega A, Galera M, Solaz E, Martinez-Hervas S, Redon J, Cortes R (2020). Decreased urinary levels of SIRT1 as non-invasive biomarker of early renal damage in hypertension. Int J Mol Sci.

[15] Morimoto M, Namba-Hamano T, Notsu S, Iwata Y, Yasuhara Y, Yamato M, Isaka Y (2023). Diabetic nephropathy with marked extra-capillary cell proliferation: a case report. BMC Nephrol.

[16] Hasegawa K (2019). Novel tubular-glomerular interplay in diabetic kidney disease mediated by sirtuin 1, nicotinamide mononucleotide, and nicotinamide adenine dinucleotide oshima award address 2017. Clin Exp Nephrol.

[17] Maor GL, Yearim A, Ast G (2015). The alternative role of DNA methylation in splicing regulation. Trends Genet.

[18] Muraoka H, Hasegawa K, Sakamaki Y, Minakuchi H, Kawaguchi T, Yasuda I, Kanda T, Tokuyama H, Wakino S, Itoh H (2019). Role of Nampt-Sirt6 axis in renal proximal tubules in extracellular matrix deposition in diabetic nephropathy. Cell Rep.

[19] Umino H, Hasegawa K, Minakuchi H, Muraoka H, Kawaguchi T, Kanda T, Tokuyama H, Wakino S, Itoh H (2018). High basolateral glucose increases sodium-glucose cotransporter 2 and reduces Sirtuin-1 in renal tubules through glucose transporter-2 detection. Sci Rep.

[20] Verissimo T, Faivre A, Rinaldi A, Lindenmeyer M, Delitsikou V, Veyrat-Durebex C, Heckenmeyer C, Fernandez M, Berchtold L, Dalga D, Cohen C, Naesens M, Ricksten SE, Martin PY, Pugin J, Merlier F, Haupt K, Rutkowski JM, Moll S, Cippà PE, Legouis D, de Seigneux S (2022). Decreased renal gluconeogenesis is a hallmark of chronic kidney disease. J Am Soc Nephrol.

[21] Hasegawa K, Sakamaki Y, Tamaki M, Wakino S (2023). PCK1 protects against mitoribosomal defects in diabetic nephropathy in mouse models. J Am Soc Nephrol.

[22] Verissimo T, Dalga D, Arnoux G, Sakhi I, Faivre A, Auwerx H, Bourgeois S, Paolucci D, Gex Q, Rutkowski JM, Legouis D, Wagner CA, Hall AM, de Seigneux S (2023). PCK1 is a key regulator of metabolic and mitochondrial functions in renal tubular cells. Am J Physiol Renal Physiol.

[23] Verissimo T, de Seigneux S (2024). New evidence of the impact of mitochondria on kidney health and disease. Nat Rev Nephrol.

[24] Wakino S, Hasegawa K, Itoh H (2015). Sirtuin and metabolic kidney disease. Kidney Int.

[25] Roehlen N, Saviano A, El Saghire H, Crouchet E, Nehme Z, Del Zompo F, Jühling F, Oudot MA, Durand SC, Duong FHT, Cherradi S, Gonzalez Motos V, Almeida N, Ponsolles C, Heydmann L, Ostyn T, Lallement A, Pessaux P, Felli E, Cavalli A, Sgrignani J, Thumann C, Koutsopoulos O, Fuchs BC, Hoshida Y, Hofmann M, Vyberg M, Viuff BM, Galsgaard ED, Elson G, Toso A, Meyer M, Iacone R, Schweighoffer T, Teixeira G, Moll S, De Vito C, Roskams T, Davidson I, Heide D, Heikenwälder M, Zeisel MB, Lupberger J, Mailly L, Schuster C, Baumert TF (2022). A monoclonal antibody targeting nonjunctional claudin-1 inhibits fibrosis in patient-derived models by modulating cell plasticity. Sci Transl Med.

[26] Baldimtsi E, Whiss PA, Wahlberg J (2023). Systemic biomarkers of microvascular alterations in type 1 diabetes associated neuropathy and nephropathy - a prospective long-term follow-up study. J Diabetes Complic.

[27] Liu M, Liang K, Zhen J, Zhou M, Wang X, Wang Z, Wei X, Zhang Y, Sun Y, Zhou Z, Su H, Zhang C, Li N, Gao C, Peng J, Yi F (2017). Sirt6 deficiency exacerbates podocyte injury and proteinuria through targeting notch signaling. Nat Commun.

[28] Perico L, Remuzzi G, Benigni A (2024). Sirtuins in kidney health and disease. Nat Rev Nephrol.

[29] Maeda S, Koya D, Araki SI, Babazono T, Umezono T, Toyoda M, Kawai K, Imanishi M, Uzu T, Suzuki D, Maegawa H, Kashiwagi A, Iwamoto Y, Nakamura Y (2011). Association between single nucleotide polymorphisms within genes encoding sirtuin families and diabetic nephropathy in Japanese subjects with type 2 diabetes. Clin Exp Nephrol.

[30] Benito-Martin A, Ucero AC, Izquierdo MC, Santamaria B, Picatoste B, Carrasco S, Lorenzo O, Ruiz-Ortega M, Egido J (1842). Ortiz A (2014) Endogenous NAMPT dampens chemokine expression and apoptotic responses in stressed tubular cells. Biochim Biophys Acta.

[31] Nomura K, Tatsumi S, Miyagawa A, Shiozaki Y, Sasaki S, Kaneko I, Ito M, Kido S, Segawa H, Sano M, Fukuwatari T, Shibata K, Miyamoto K (2014). Hepatectomy-related hypophosphatemia: a novel phosphaturic factor in the liver-kidney axis. J Am Soc Nephrol.

[32] Jia Y, Kang X, Tan L, Ren Y, Qu L, Tang J, Liu G, Wang S, Xiong Z, Yang L (2021). Nicotinamide mononucleotide attenuates renal interstitial fibrosis after AKI by suppressing tubular DNA damage and senescence. Front Physiol.

[33] Huang P, Zhou Y, Tang W, Ren C, Jiang A, Wang X, Qian X, Zhou Z, Gong A (2022). Long-term treatment of nicotinamide mononucleotide improved age-related diminished ovary reserve through enhancing the mitophagy level of granulosa cells in mice. J Nutr Biochem.

[34] Mills KF, Yoshida S, Stein LR, Grozio A, Kubota S, Sasaki Y, Redpath P, Migaud ME, Apte RS, Uchida K, Yoshino J, Imai SI (2016). Long-term administration of nicotinamide mononucleotide mitigates age-associated physiological decline in mice. Cell Metab.

