# Correction to: Ability of NAD and Sirt1 to epigenetically suppress albuminuria

**DOI:** 10.1007/s10157-024-02523-5

**Published:** 2024-06-25

**Authors:** Kazuhiro Hasegawa, Masanori Tamaki, Eriko Shibata, Taizo Inagaki, Masanori Minato, Sumiyo Yamaguchi, Ikuko Shimizu, Shinji Miyakami, Miho Tada, Shu Wakino

**Affiliations:** Department of Nephrology, Tokushima University Graduate School of Biomedical Sciences, 3-18-15 Kuramoto-cho, Tokushima, 770-8503 Japan

**Correction to: Clinical and Experimental Nephrology** 10.1007/s10157-024-02502-w

The article “Ability of NAD and Sirt1 to epigenetically suppress albuminuria”, written by Kazuhiro Hasegawa · Masanori Tamaki · Eriko Shibata · Taizo Inagaki · Masanori Minato · Sumiyo Yamaguchi ·Ikuko Shimizu · Shinji Miyakami · Miho Tada · Shu Wakino was originally published Online First without Open Access. With the author(s)’ decision to opt for Open Choice the copyright of the article changed on 27th May 2024 to © The Author(s) 2024 and the article is forthwith distributed under the terms of the Creative Commons Attribution 4.0 International License (https://creativecommons.org/licenses/by/4.0/), which permits use, sharing, adaptation, distribution and reproduction in any medium or format, as long as you give appropriate credit to the original author(s) and the source, provide a link to the Creative Commons licence, and indicate if changes were made.

The original article has been corrected.

